# Supplementary material for: A common neuronal ensemble in nucleus accumbens regulates pain-like behaviour and sleep
Source: Nat Commun. 2023 Aug 5;14:4700. doi: 10.1038/s41467-023-40450-3 (PMC10404280; doi:10.1038/s41467-023-40450-3)
Supplement: Supplementary file 1 — Supplementary Information [file 41467_2023_40450_MOESM1_ESM.pdf]

Supplementary Materials

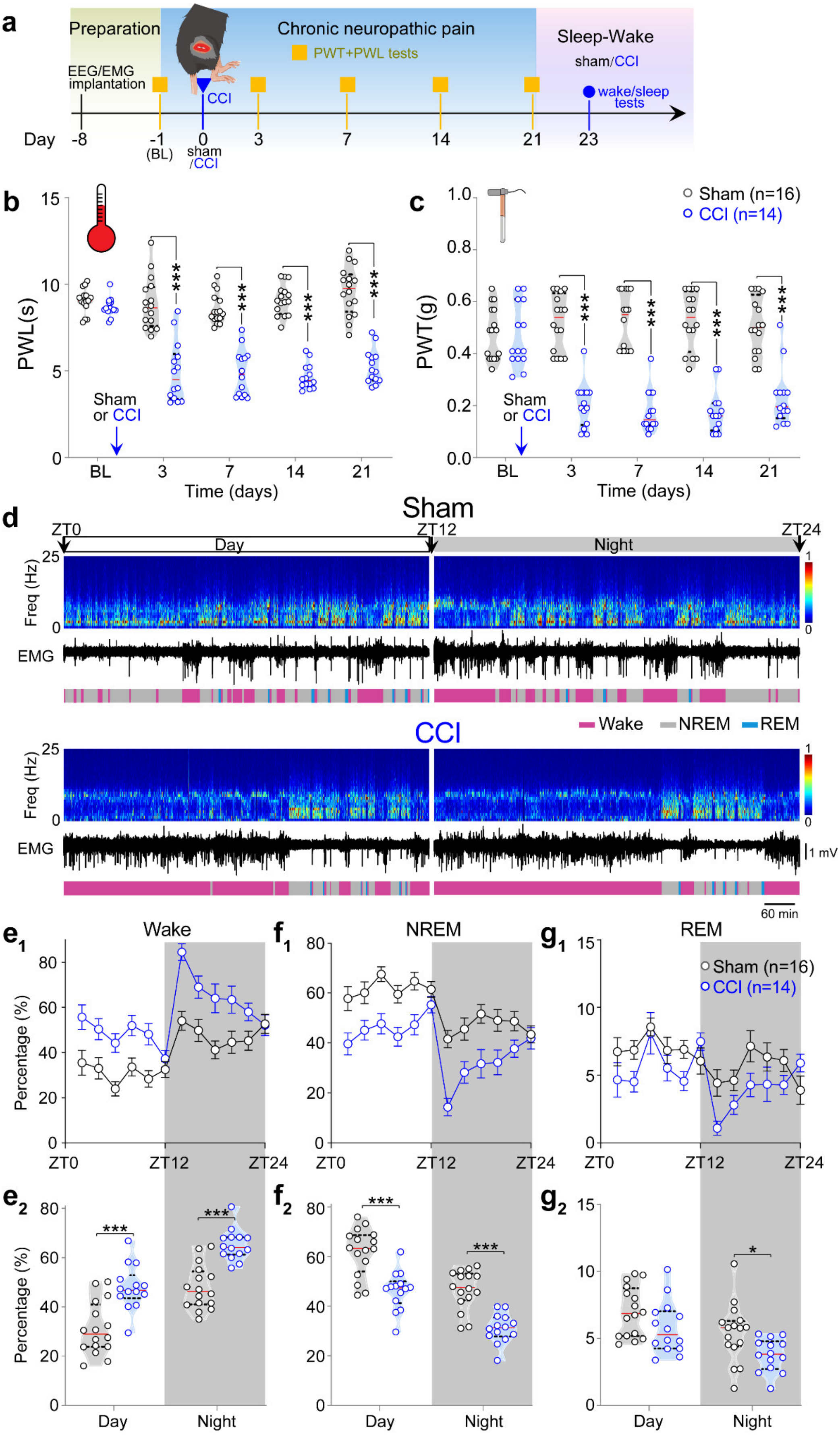

Supplementary Fig. 1 Chronic pain causes sleep loss.

a Experimental scheme of EEG/EMG implantation, sham/CCI surgery, PWT/PWL assessment, and EEG/EMG recording in C57BL/6J mice. PWT and PWL were assessed at day 1 before, and at days 3, 7, 14 and 21 after the sham or CCI surgery. EEG/EMG recordings were conducted from day 23 after the CCI surgery. **b, c** Violin plots showing that compared with the sham mice (black, n = 16 mice), CCI mice (red, n = 14 mice) exhibited decreased PWL (Group, $F_{(1,28)} = 332.2$, $p < 0.001$, two-way ANOVA; sham vs. CCI, days 3: $p < 0.001$, days 7: $p < 0.001$, days 14: $p < 0.001$, days 21: $p < 0.001$, Bonferroni's multiple comparisons test) and PWT (Group, $F_{(1,28)} = 146.4$, $p < 0.001$, two-way ANOVA; sham vs. CCI, days 3: $p < 0.001$, days 7: $p < 0.001$, days 14: $p < 0.001$, days 21: $p < 0.001$, Bonferroni's multiple comparisons test) in the injured paw at days 3, 7, 14 and 21 following the CCI surgery. In this and the following violin plots, data are presented as median (red line) with 25th and 75th percentile (dash line). **d** 24 hours continuous EEG spectrogram, EMG trace, and brain states (color-coded) data recorded from sham and CCI mice. Freq., frequency. **e_1-g_1** The graphs illustrate the average (mean ± SEM) percentages of wake, NREM, or REM sleep during the day and night for sham (black, n = 16 mice) and CCI mice (blue, n = 14 mice). **e_2-g_2** Comparison of the percentages of time in each brain state during the day and night for sham and CCI mice. CCI mice exhibited significantly more wakefulness (Group, $F_{(1,28)} = 46$, $p < 0.001$, two-way ANOVA; sham vs. CCI, day $p < 0.001$, night $p < 0.001$, Bonferroni's multiple comparisons test), less NREM (Group, $F_{(1,28)} = 45.18$, $p < 0.001$, two-way ANOVA; sham vs. CCI, day: $p < 0.001$, night: $p < 0.001$, Bonferroni's multiple comparisons test), and REM sleep (Group, $F_{(1,28)} = 8.048$, $p = 0.008$, two-way ANOVA; sham vs. CCI, day $p = 0.174$, night $p = 0.039$, Bonferroni's multiple comparisons test) during the day and night. * $p < 0.05$; ** $p < 0.01$; *** $p < 0.001$ (two-way ANOVA with Bonferroni's multiple comparisons test). *** $p < 0.001$. Two-way ANOVA test with Bonferroni's multiple comparisons test for (**b, c, e_2, f_2, g_2.**).

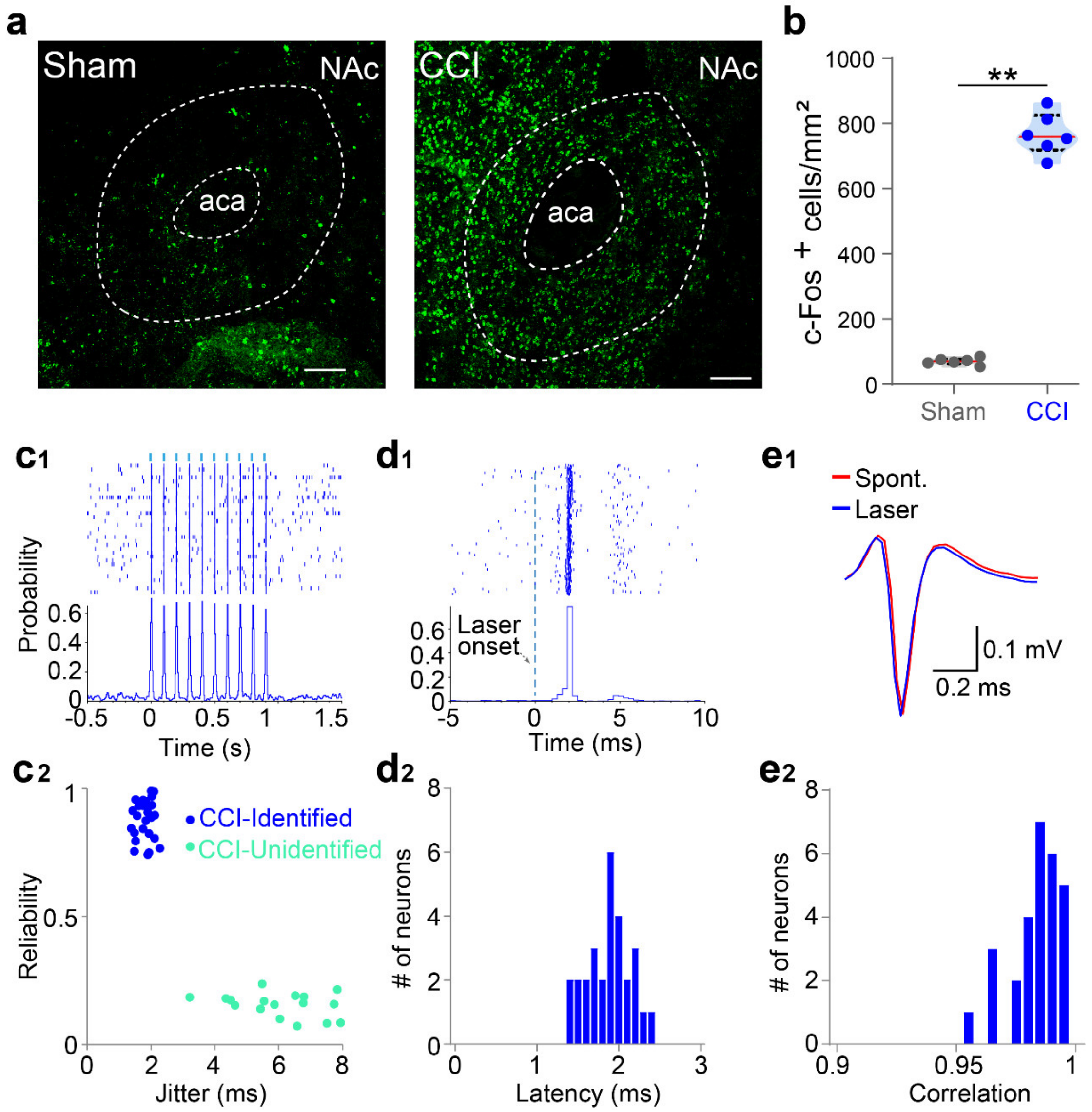

Supplementary Fig. 2 The c-Fos expression in NAc of sham or CCI mice and optogenetic identification of NAc ensemble.

a FISH showing the c-Fos expression in NAc three weeks after sham and CCI surgery respectively. Scale bar: 200 µm. **b** Statistical analysis of c-Fos expression in NAc under different conditions as in (a). ** p = 0.0022 versus the c-Fos+ in sham mice (sham, n = 6 mice; CCI, n = 6 mice, Two-tailed Mann Whitney test). **c_1** Representative peri-event raster plots (top) and peri-stimulus time histogram (PSTH, bottom) showing the firing activity of an identified NAc ensemble before, during, and after 10 Hz laser stimulation. Cyan ticks indicate laser pulses. **c_2** Reliability vs. temporal jitter of laser-evoked spikes indicating that high reliability, low jitter for identified NAc ensemble (blue), and low

reliability, diverse jitters for unidentified neurons (cyan). **d$_1$** Sample of peri-event raster plots (top) and PSTH (bottom) for laser-evoked spikes of a ChR2-tagged NAc ensemble. The dashed line indicates laser onset time. **d$_2$** Plot of latency distribution showing short latencies of laser-evoked spiking for all ChR2-tagged NAc ensemble. **e$_1$** Representative waveforms (averaged) of laser-evoked spikes (blue) and spontaneous spikes (red) of an identified NAc ensemble neuron. **e$_2$** Distribution plot showing high correlation coefficients between laser-evoked and spontaneous spike waveforms for all identified NAc ensemble.

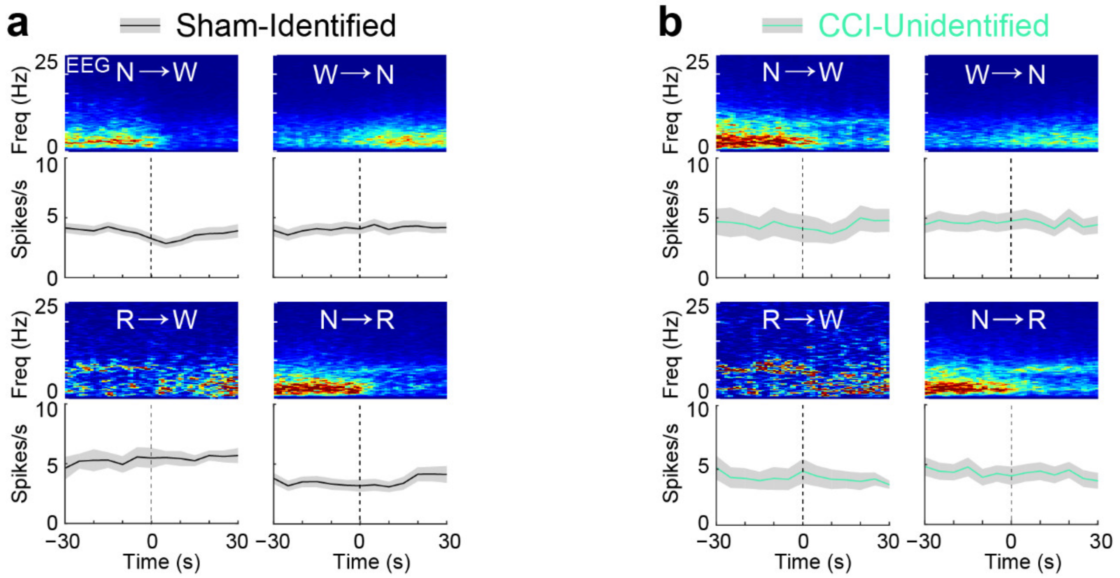

Supplementary Fig. 3 No obvious change in the firing rate of identified NAc neurons in sham mice and unidentified NAc neurons in CCI mice during the transitions between two different brain states.

a Mean firing rates of identified NAc neurons in sham mice (black, n = 34 neurons, from 7 mice) during different brain state transitions: NREM-to-wake (top left), wake-to-NREM (top right), REM-to-wake (bottom left), and NREM-to-REM (bottom right). Note that no obvious change in the firing rate during different brain state transitions. Top inset: average EEG spectrogram 30 s before and after each transition. Down inset: firing rate at the transition averaged across all 34 NAc neurons in sham mice. Time 0 indicates the transition point. Shading represents ± SEM. **b** Similar to panel **a**, but for

unidentified NAc neurons in CCI mice (cyan, n = 17 neurons, from 6 mice).

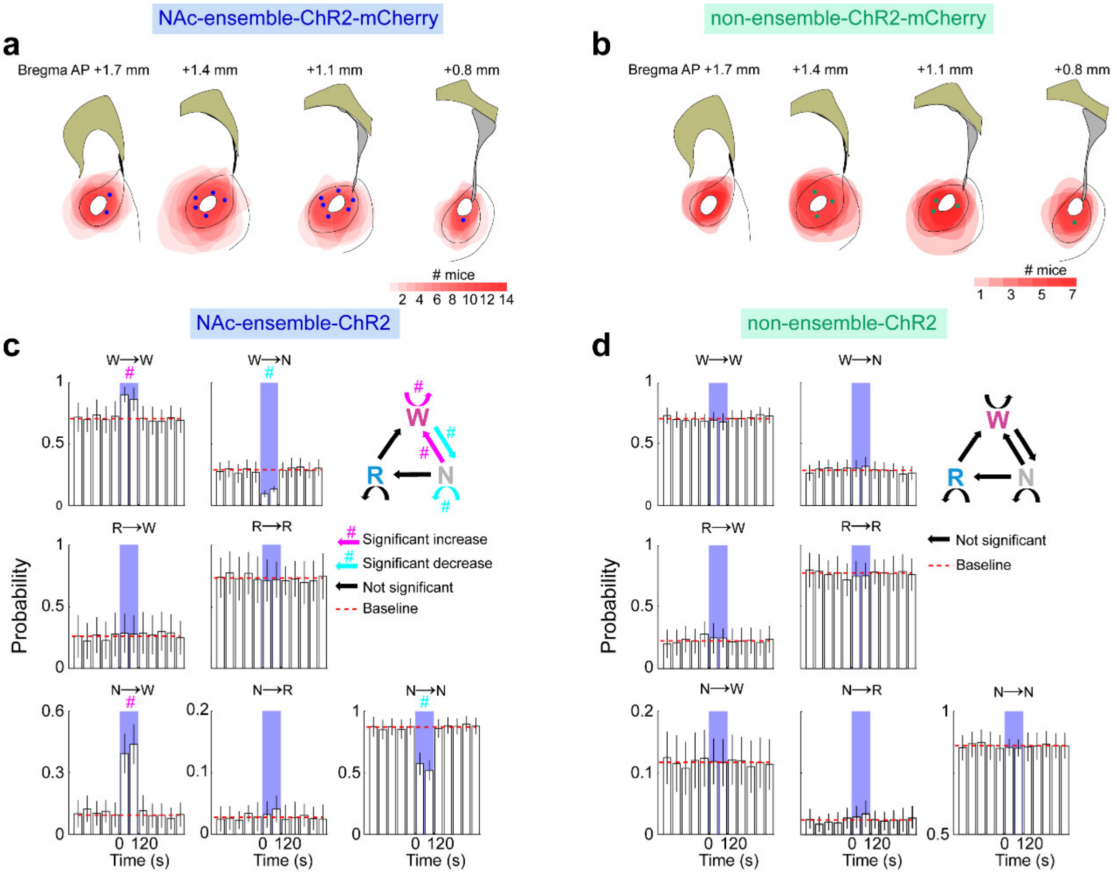

Supplementary Fig. 4 The expression for ChR2-mCherry in NAc of NAc-ensemble-ChR2 and non-ensemble-ChR2 mice, the effect of activating NAc ensemble and non-ensemble on the transition probability of brain states.

a, b Average distribution of ChR2-mCherry fluorescence expression in NAc-ensemble-ChR2 (A) and non-ensemble-ChR2 (B) mice. For each mouse (n = 14 NAc-ensemble-ChR2 mice, 7 non-ensemble-ChR2 mice), we chose 4 coronal brain sections to determine the spread of ChR2-mCherry (from +1.7 mm to +0.8 mm along the rostrocaudal axis, where most of the virus expression was observed). Blue and green dots represent the positions of optic fiber tips in NAc-ensemble-ChR2 and non-ensemble-ChR2 mice respectively. Brain figures were adapted from Allen Mouse Brain Atlas (2011 Allen Institute for Cell Science, Allen Mouse Brain Atlas, available at http://atlas.brain-map.org/). **c, d** 10Hz laser stimulation (blue shading, 10 Hz, 473 nm, 120 s) caused the changes of transition probability between each pair of brain states in

NAc-ensemble-ChR2 mice (n = 14 mice), but not in non-ensemble-ChR2 mice (n = 7 mice). The diagram summarizes transitions that are significantly increased (magenta), decreased (cyan), or unaffected (black) by laser stimulation. Magenta and cyan asterisk (#) indicates significant increase and decrease in transition probability during laser stimulation compared to baseline (wake→wake, p = 0.012; wake→NREM, p = 0.013; NREM→wake, p = 0.011; NREM→NREM, p = 0.012; bootstrap). Bars, means of transition probabilities within each 60 s period. Error bar, 95% CI (bootstrap).

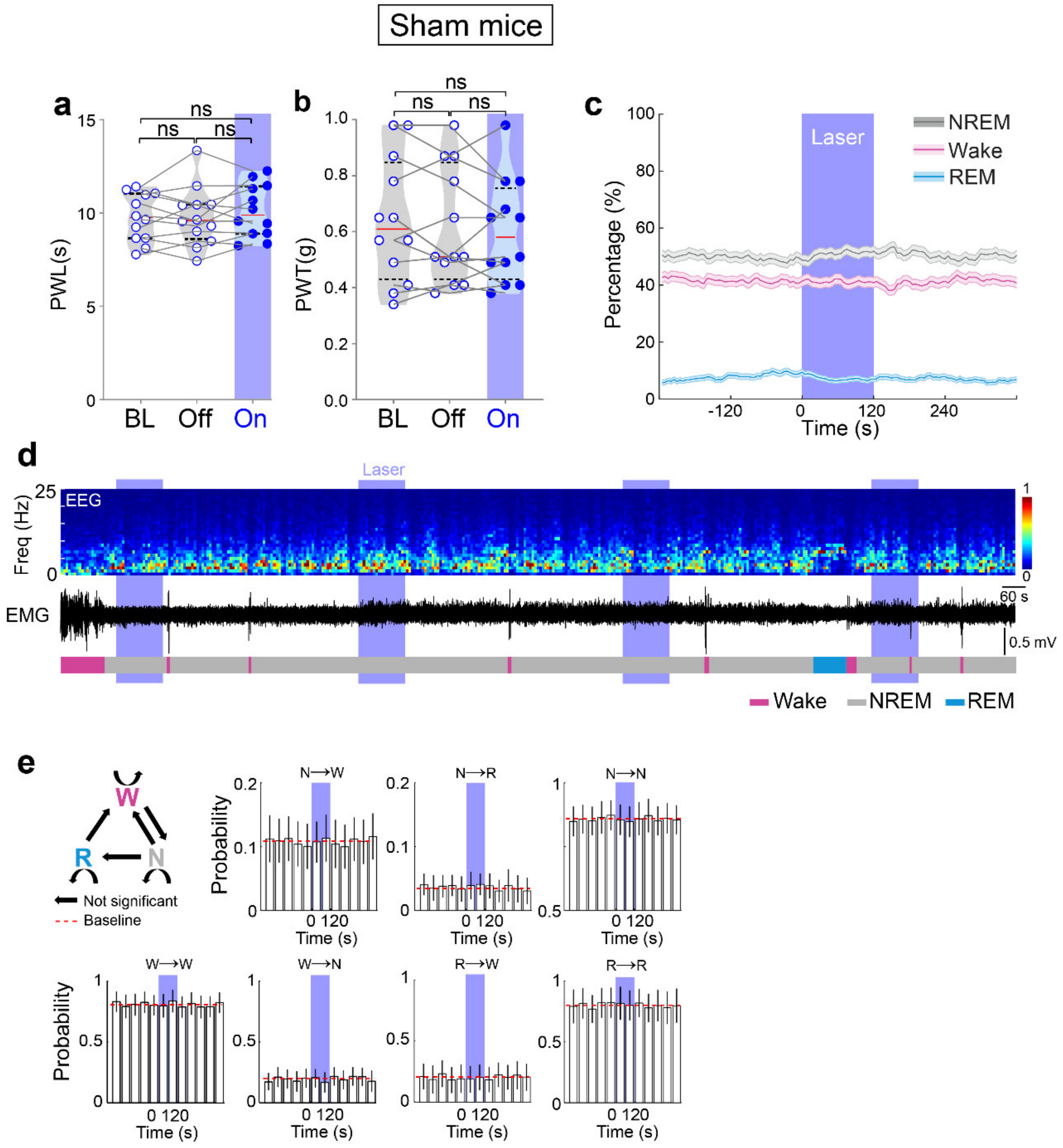

Supplementary Fig. 5 No effect of activating ChR2-expressing NAc neurons in sham mice on pain thresholds and sleep-wake behavior.

Activation of ChR2-expressing NAc neurons in c-fos-tTA transgenic mice underwent sham surgery. **a, b** No significant differences in thermal pain threshold (PWL, n = 12 mice, $F_{(1.98, 20.8)} = 0.962$, p = 0.397, Repeated measures one-way ANOVA test; BL vs. Off, p > 0.99, BL vs. On, p = 0.68, Off vs. On, p = 0.93, Bonferroni's multiple comparisons test) and mechanical pain threshold (PWT, n = 12 mice, $F_{(1.86, 20.5)}$ = 0.909, p = 0.412, Repeated measures one-way ANOVA test; BL vs. Off, p = 0.994, BL vs. On, p = 0.729, Off vs. On, p > 0.99, Bonferroni's multiple comparisons test) are observed in sham mice between with and without laser activations. ns, not significant. **c** No effect of 10Hz blue laser stimulation on brain states in sham mice (n = 12 mice, p = 0.291, = 0.423, and = 0.310 for NREM, wake, and REM states). Data are mean ± SEM. Shading represents ± SEM. **d** An example of optogenetic activation of ChR2-expressing NAc neurons in sham mice. Shown are an EEG spectrogram (top), EMG trace (middle), and brain states (bottom). Blue stripe indicates laser stimulation (10 Hz, 120 s). **e** No effect of laser stimulation on transition probability between each pair of brain states in sham mice (n = 12 mice). Bars, means of transition probabilities within each 60 s period. Error bar, 95% CI (bootstrap).

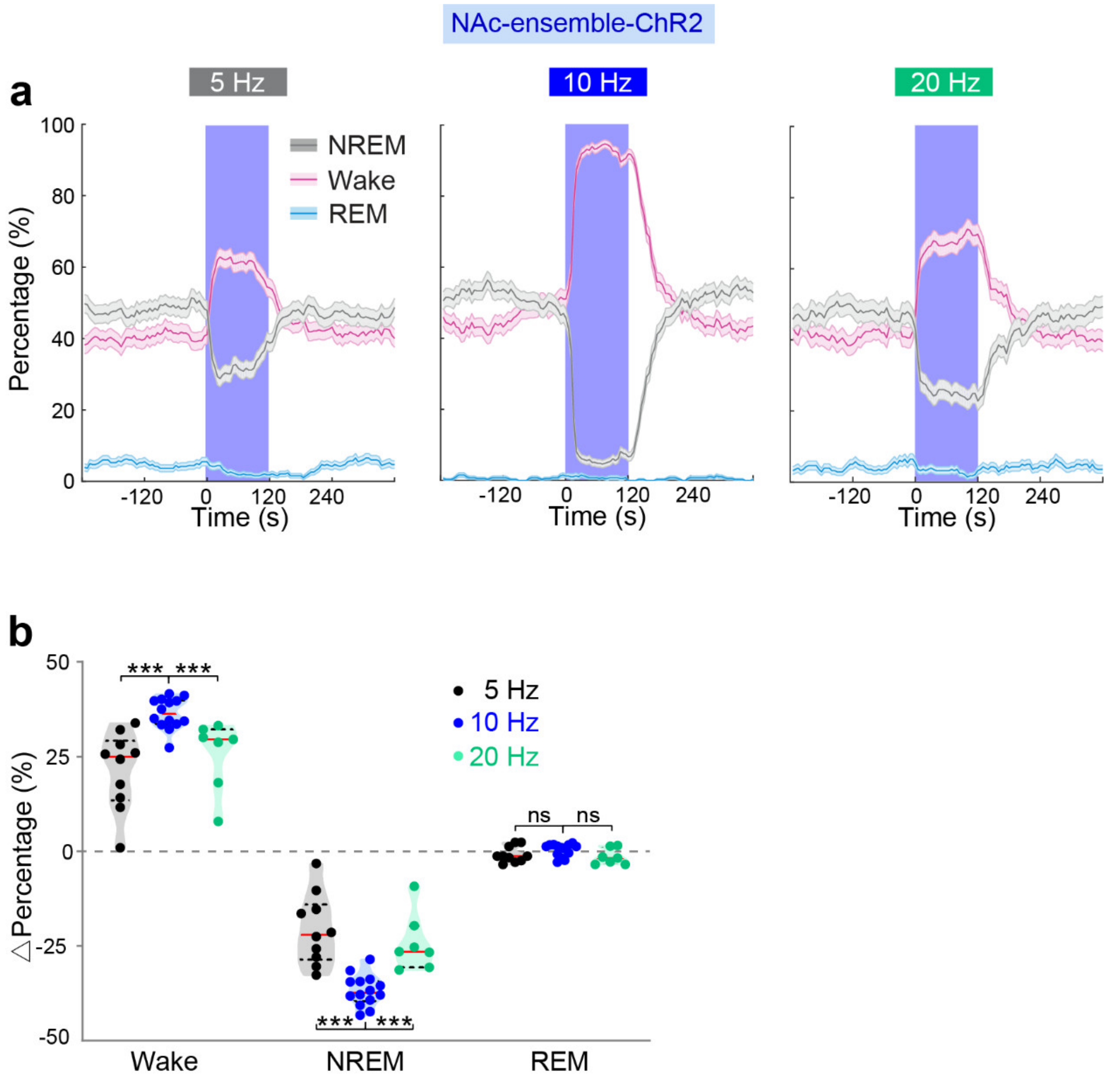

Supplementary Fig. 6 Effect of activating NAc ensemble by blue laser stimulations at 5 Hz, 10Hz, and 20Hz.

a Effect of activating NAc ensemble by blue laser stimulations at 5 Hz (left: gray, n = 10mice), 10 Hz (middle: blue, n = 14 mice), and 20 Hz (right: green, n = 7 mice). Data are mean ± SEM. Shading represents ± SEM. The same experimental design as in Fig. **2a**, NAc ensemble were labeled and activated in c-fos-tTA transgenic mice underwent CCI surgery. **b** Changes in the percentage of each brain state (difference between the 240 s period before and 120 s during laser stimulation) induced by ChR2-mediated activation of NAc ensemble (5 Hz: black, n = 10 mice; 10 Hz: blue, n = 14 mice; and 20 Hz: green, n = 7 mice). Note a significantly large-magnitude of wakefulness increase and NREM decrease induced by blue laser stimulation at 10 Hz.

*** $p < 0.001$ versus 10 Hz laser stimulation (Group, $F_{(2, 28)} = 14.69$, $p < 0.001$, two-way ANOVA; wake: 5 Hz or 20 Hz vs. 10 Hz, $p < 0.001$; NREM: 5 Hz or 20 Hz vs. 10 Hz, $p < 0.001$; REM: 5 Hz or 20 Hz vs. 10 Hz, $p > 0.05$; Bonferroni's multiple comparisons test). ns, not significant.

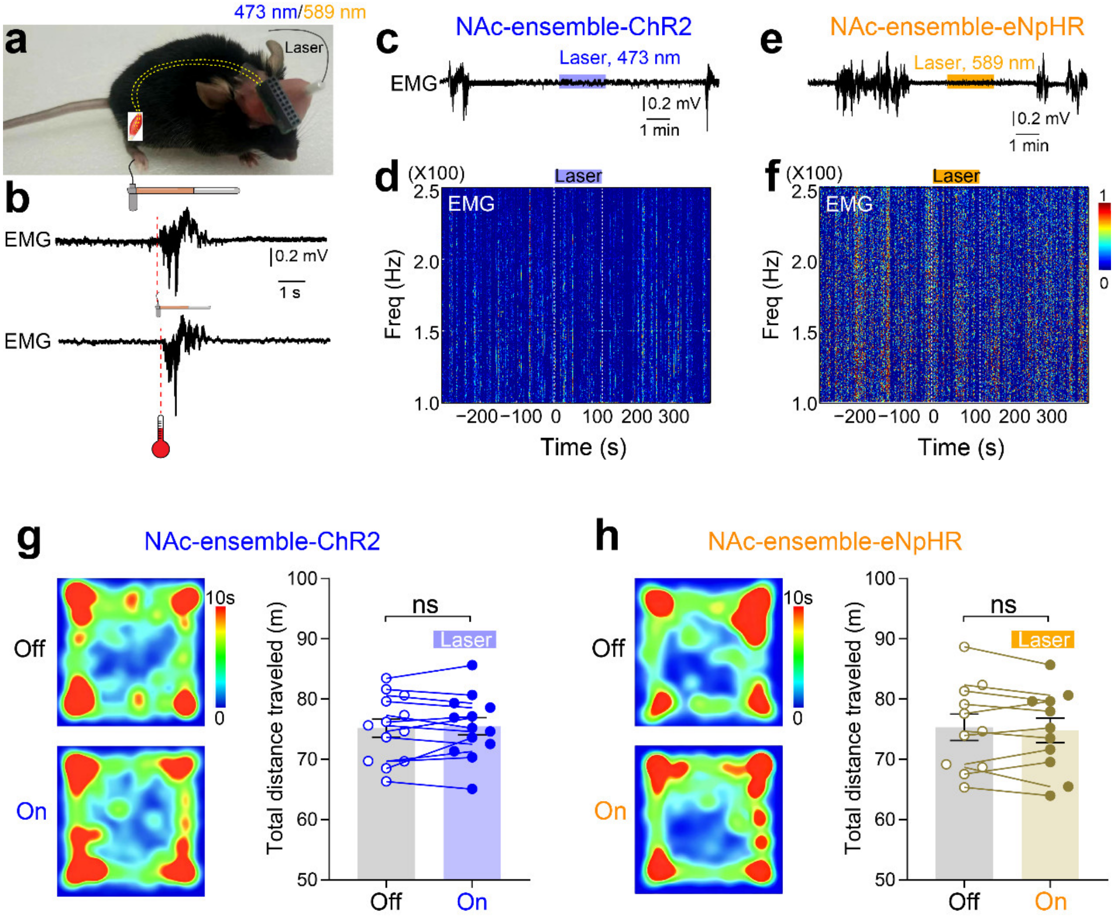

Supplementary Fig. 7 Optogenetic stimulations of NAc ensemble do not affect the movement of CCI-hindlimb in EMG recording and locomotion behavior in open field test.

a Illustration of intramuscular electrode implantation on medial gastrocnemius muscles of CCI-hindlimb and the head connector. Dash lines represented the EMG cables. **b** Representative traces showing EMG recording of hindlimb muscles in a CCI mouse before, during, and after a 0.07-g von Frey filament stimuli (top) and thermal stimuli (bottom). **c** EMG trace recording of hindlimb muscles from a CCI mouse during baseline conditions and 10 Hz blue laser (473 nm, 120 s) activation of NAc ensemble

neurons. **d** Normalized EMG spectrogram aligned to blue laser stimulation time (During 3-hour recording, blue laser stimulations were delivered randomly from a uniform distribution between 4 and 10 min). Note no significant change in EMG spectral power upon blue laser stimulation. **e** EMG trace recording of hindlimb muscles from a CCI mouse during baseline conditions and yellow laser (589 nm, 8 s on/2 s off, 120 s) inactivation of NAc ensemble neurons. **f** Normalized EMG spectrogram aligned to yellow laser stimulation time (During 3-hour recording, yellow laser stimulations were delivered randomly from a uniform distribution between 4 and 10 min). Note no obvious change in EMG spectral power upon yellow laser stimulation. **g** Left: Representative heat maps of NAc-ensemble-ChR2 mice within the open field box over a 10-minute period in the absence or presence of blue laser activation. Right: Optogenetic activation of NAc ensemble did not change the total distance traveled by NAc-ensemble-ChR2 mice in the open field test (n = 13 mice, mean ± SEM, Off vs On: $t = 0.543$, df = 12, $P = 0.60$, Two-tailed paired t test). **h** Left: Representative heat maps of NAc-ensemble-eNpHR mice within the open field box over a 10-minute period in the absence or presence of yellow laser inactivation. Right: Optogenetic inactivation of NAc ensemble did not change the total distance traveled by NAc-ensemble- eNpHR mice in the open field test (n = 11 mice, mean ± SEM, Off vs On: $t = 0.816$, df = 10, $P = 0.43$, Two-tailed paired t test). ns, not significant. Two-tailed paired t test for (**g, h**).

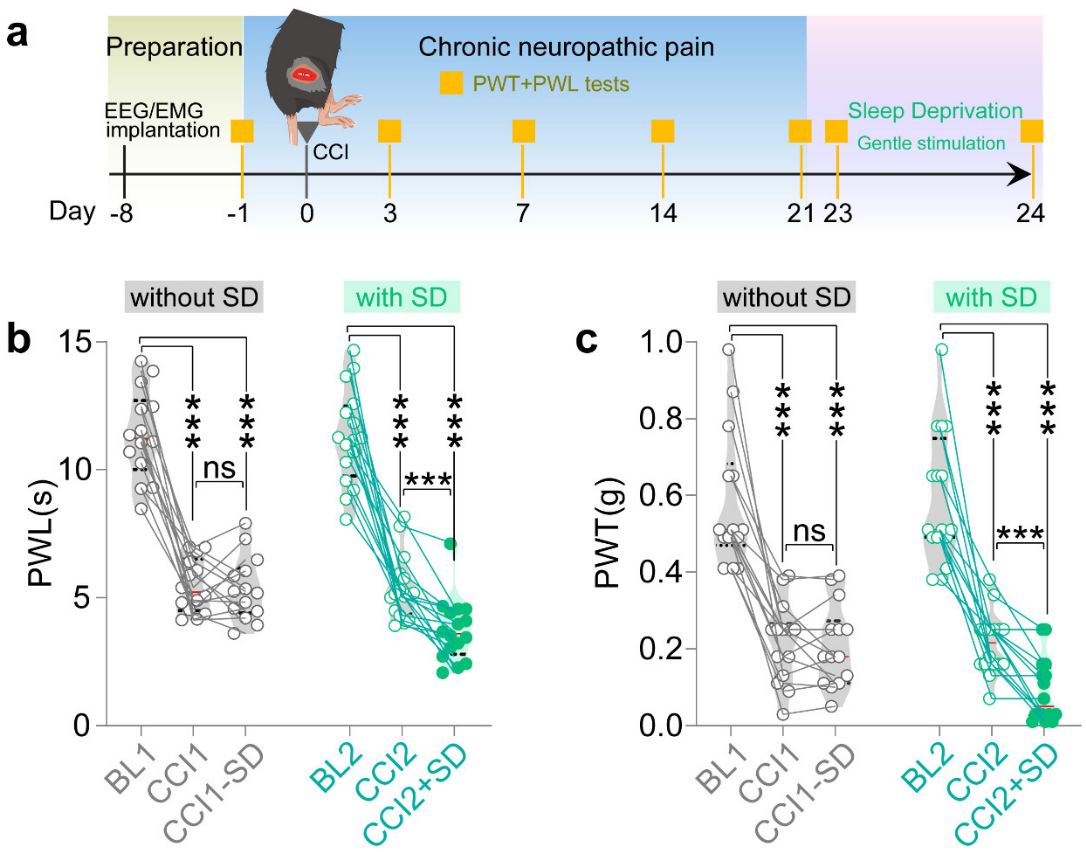

Supplementary Fig. 8 Sleep deprivation exacerbates chronic neuropathic pain.

a Experimental scheme of EEG/EMG implantation, CCI surgery, PWT/PWL assessment, and sleep deprivation in C57BL/6J mice. PWT and PWL were assessed at day 1 before, at days 3, 7, 14, 21 after the CCI surgery, and before/after sleep deprivation. 6 hours sleep deprivation were performed 23 days after the CCI surgery. **b, c** Violin plots showing that sleep deprivation significantly decreased thermal PWL (n = 16 mice, $F_{(1.37, 20.5)} = 124$, $p < 0.0001$, Repeated measures one-way ANOVA test; BL2 vs. CCI2, $p < 0.001$, BL2 vs. CCI2+SD, $p < 0.001$, CCI2 vs. CCI2+SD, $p < 0.001$, Bonferroni's multiple comparisons test) and mechanical PWT (n = 16 mice, $F_{(1.20, 17.9)} = 75.0$, $p < 0.0001$, Repeated measures one-way ANOVA test; BL2 vs. CCI2, $p < 0.001$, BL2 vs. CCI2+SD, $p < 0.001$, CCI2 vs. CCI2+SD, $p < 0.001$, Bonferroni's multiple comparisons test) in sleep-deprived CCI mice. Both PWL (n = 14 mice, $F_{(1.49, 19.3)} = 89.6$, $p < 0.0001$, Repeated measures one-way ANOVA test; BL1 vs. CCI1, $p < 0.001$, BL1 vs. CCI1-SD, $p < 0.001$, CCI1 vs. CCI1-SD, $p > 0.99$, Bonferroni's multiple comparisons test) and PWT (n = 14 mice, $F_{(1.19, 15.5)} = 51.2$, $p < 0.0001$, Repeated measures one-way ANOVA test; BL1 vs. CCI1, $p < 0.001$, BL1 vs. CCI1-SD, $p < 0.001$,

CCI1 vs. CCI1-SD, p > 0.99, Bonferroni's multiple comparisons test) did not change much in CCI mice without sleep deprivation. *** $p < 0.001$, ns, not significant. Repeated measures one-way ANOVA test with Bonferroni's multiple comparisons test for (**b, c**).

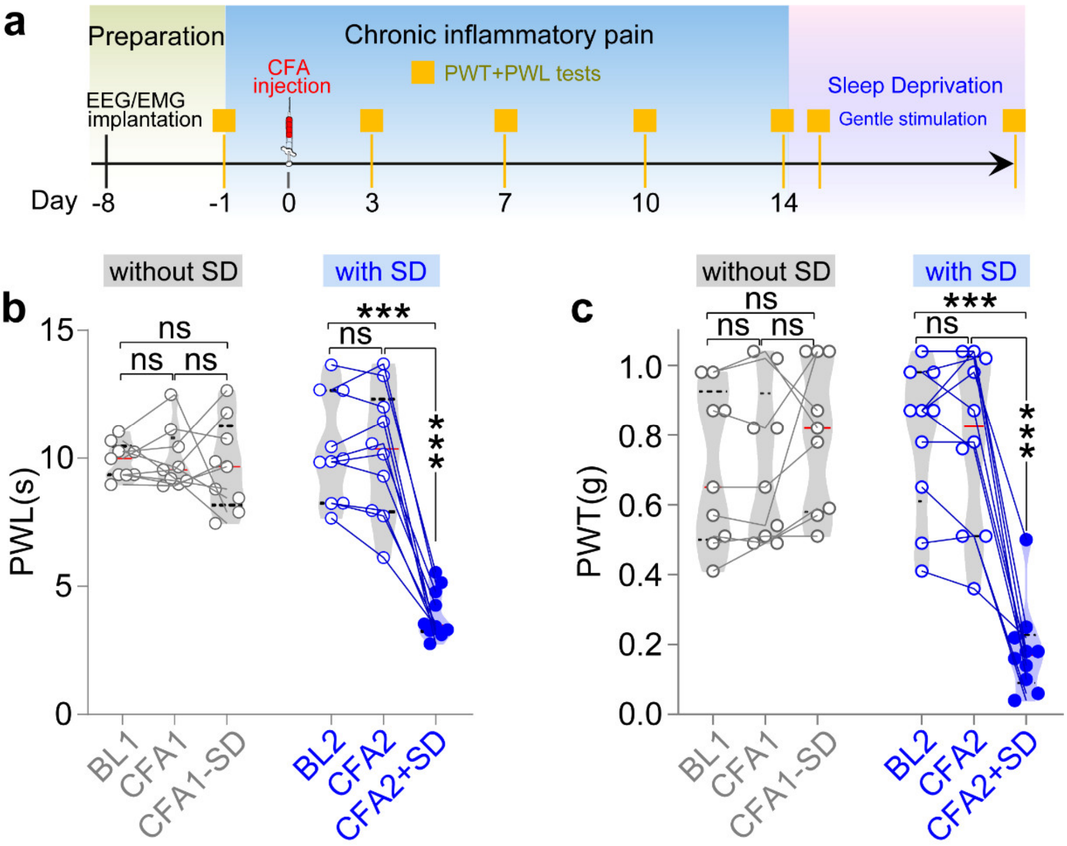

Supplementary Fig. 9 Sleep deprivation induces hyperalgesia in CFA mice recovered from chronic inflammatory pain.

a Experimental scheme of EEG/EMG implantation, CFA injection, PWT/PWL assessment, and sleep deprivation in C57BL/6J mice. PWT and PWL were assessed at day 1 before, at days 3, 7, 10, 14 after CFA injection, and before/after sleep deprivation. 6 hours sleep deprivation were performed 14 days after CFA injection. **b, c** Violin plots showing that sleep deprivation significantly decreased thermal PWL (n = 10 mice, $F_{(1.17, 10.6)} = 95$, $p < 0.0001$, Repeated measures one-way ANOVA test; BL2 vs. CFA2, $p > 0.99$, BL2 vs. CFA2+SD, $p < 0.0001$, CFA2 vs. CFA2+SD, $p < 0.0001$, Bonferroni's multiple comparisons test) and mechanical PWT (n = 10 mice, $F_{(1.29, 11.6)} = 64.3$, p <

0.0001, Repeated measures one-way ANOVA test; BL2 vs. CFA2, p > 0.99, BL2 vs. CFA2+SD, p < 0.0001, CFA2 vs. CFA2+SD, p < 0.0001, Bonferroni's multiple comparisons test) in sleep-deprived CFA mice recovered from chronic inflammatory pain. Both PWL (n = 9 mice, $F_{(1.26, 10.1)}$ = 0.09, p = 0.82, Repeated measures one-way ANOVA test; BL1 vs. CFA1, p > 0.99, BL1 vs. CFA1-SD, p > 0.99, CFA1 vs. CFA1-SD, p > 0.99, Bonferroni's multiple comparisons test) and PWT (n = 9 mice, $F_{(1.05, 8.42)}$ = 2.22, p = 0.17, Repeated measures one-way ANOVA test; BL1 vs. CFA1, p > 0.99, BL1 vs. CFA1-SD, p = 0.4, CFA1 vs. CFA1-SD, p > 0.62, Bonferroni's multiple comparisons test) did not change much in CFA mice without sleep deprivation. *** p < 0.001, ns, not significant. Repeated measures one-way ANOVA test with Bonferroni's multiple comparisons test for (**b, c**).

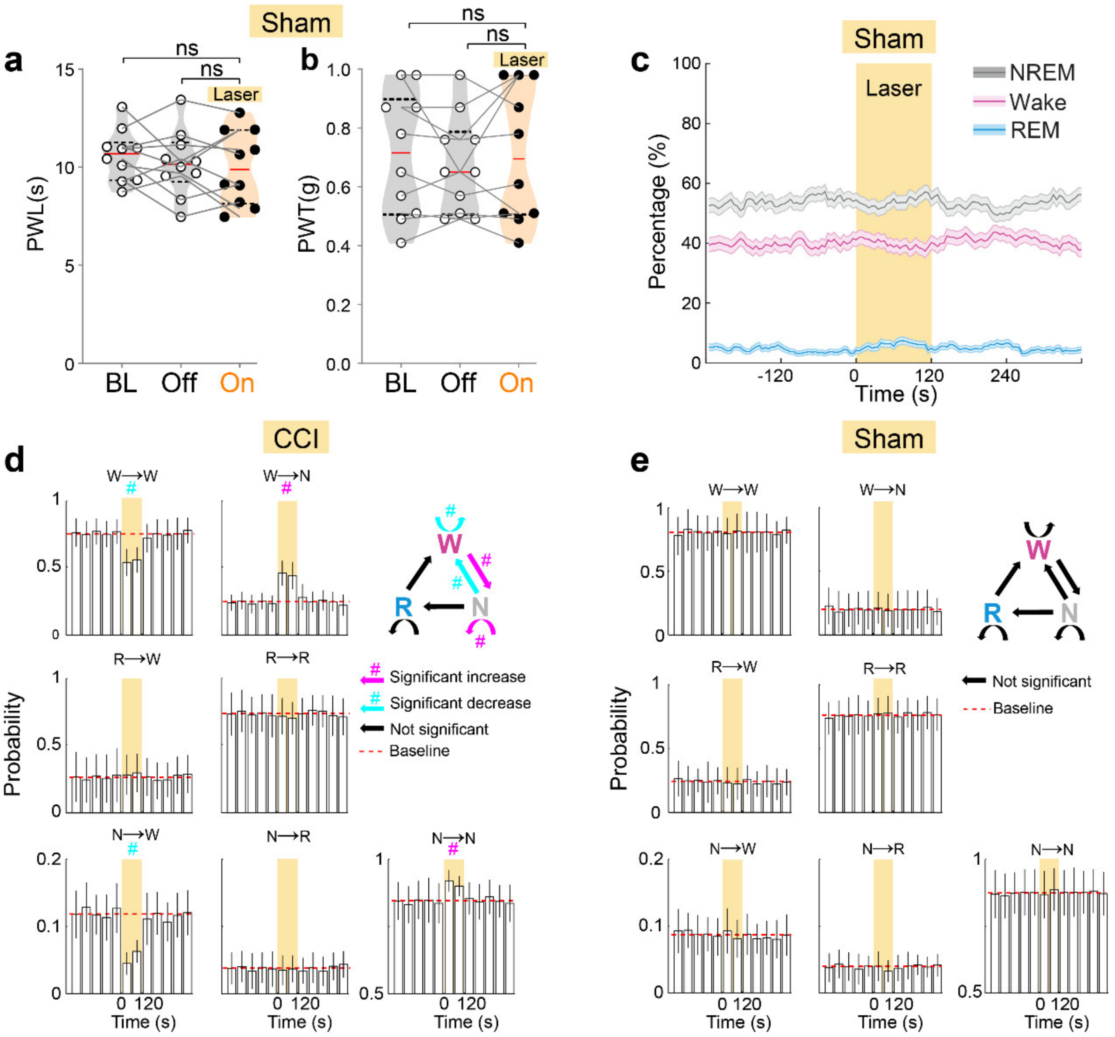

Supplementary Fig. 10 No effect of inactivating eNpHR-expressing NAc neurons in sham mice on pain thresholds and sleep-wake behavior, and optogenetic inhibition of NAc ensemble causes the changes of brain states transition in CCI mice.

a, **b** No significant differences in thermal pain latency (PWL, n = 10 mice, $F_{(1.30, 11.7)} = 0.62$, p = 0.444, Repeated measures one-way ANOVA test; BL vs. On, p > 0.99, Off vs. On, p > 0.99, Bonferroni's multiple comparisons test) and mechanical pain threshold (PWT, n = 10 mice, $F_{(1.68, 15.1)} = 1.07$, p = 0.356, Repeated measures one-way ANOVA test; BL vs. On, p = 0.264, Off vs. On, p > 0.99, Bonferroni's multiple comparisons test) are observed in sham mice between with and without laser inactivations. ns, not significant. **c** No effect of yellow laser stimulation (589 nm, yellow laser, 8 s on/2 s off, 120 s) on brain states in sham mice (n = 10 mice, p = 0.414, = 0.460, and = 0.084 for NREM, wake, and REM states). Data are mean ± SEM. Shading represents ± SEM. **d** Yellow laser inactivation of NAc ensemble (yellow shading, 589 nm, 8 s on/2 s off,120 s) caused the changes of transition probability between each pair of brain states in CCI mice (n =11). The diagram summarizes transitions that are significantly increased (magenta), decreased (cyan), or unaffected (black) by laser stimulation. Magenta and cyan asterisk (#) indicates significant increase and decrease in transition probability during laser stimulation compared to baseline (wake→wake, p = 0.024; wake→NREM, p = 0.032; NREM→wake, p = 0.026; NREM→NREM, p = 0.012; bootstrap). Bars, means of transition probabilities within each 60 s period. Error bar, 95% CI (bootstrap). **e** No effect of laser stimulation on transition probability between each pair of brain states in sham mice (n = 10 mice). ns, not significant. Bars, means of transition probabilities within each 60 s period. Error bar, 95% CI (bootstrap). Repeated measures one-way ANOVA test with Bonferroni's multiple comparisons test for (**a, b**).

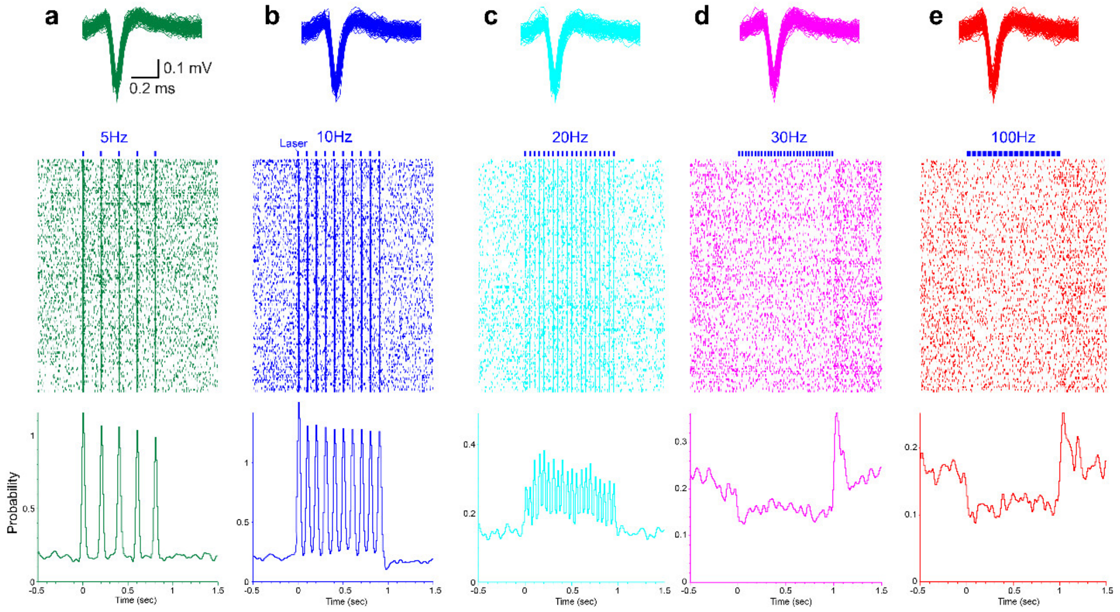

Supplementary Fig. 11 Frequency-dependent responses of firing of NAc D1 neurons.

a-e Raster plot (top) and PSTH (bottom) of the NAc D1 unit as in showing the firing activity before, during, and after 1 s optical stimulation (5Hz, 10Hz, 20Hz, 30Hz, and 100Hz), the duration of individual laser pulse is 5 ms. Blue ticks indicate 473 nm laser pulses.

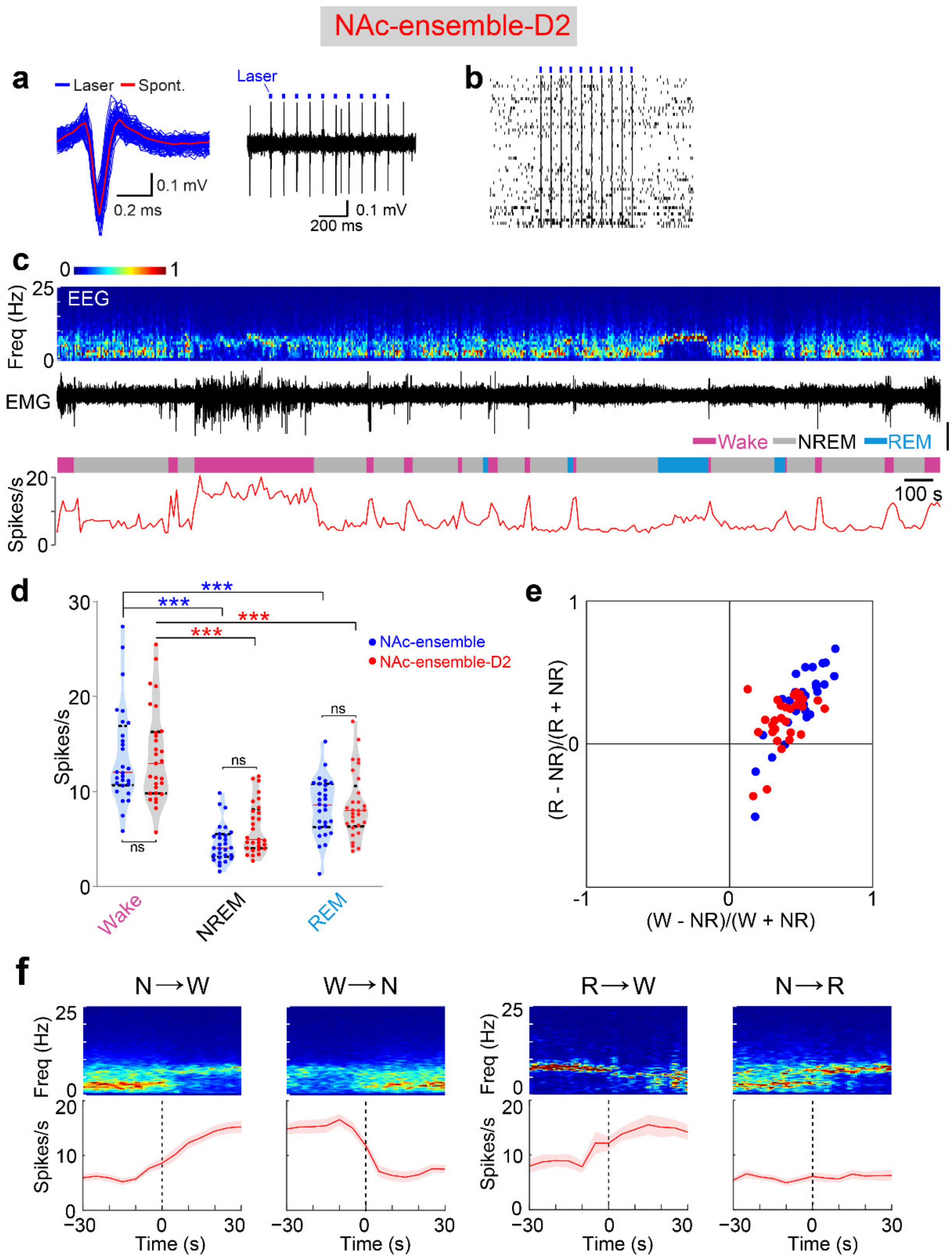

Supplementary Fig. 12 NAc-ensemble-D2 Neurons are preferentially active during wakefulness.

a Waveforms of average spontaneous (red) and individual laser-evoked (blue) spikes from an identified NAc-ensemble-D2 neuron in the NAc ensemble. **b** Example of raster plot from a NAc-ensemble-D2 neuron showing consecutive laser stimulation trials at 10 Hz. **c** Representative firing rate of a NAc-ensemble-D2 neuron (bottom) together with brain states (color-coded), EMG trace (middle), and EEG spectrogram (top). **d**

Violin plot displaying the individual firing rates of identified NAc-ensemble neurons (blue, n = 28 units from 6 mice, the same data as in Fig. 1k) and NAc-ensemble-D2 neurons in CCI mice (red, n = 29 units from 5 mice) during different brain states. *** $p < 0.001$ (State, $F_{(2,110)} = 158.7$, $p < 0.001$, two-way ANOVA; wake vs. NREM, $p < 0.001$; wake vs. REM, $p < 0.001$, Bonferroni's multiple comparisons test). ns: not significant, versus the NAc-ensemble neurons during different brain states (Group, $F_{(1,55)} = 0.557$, $p = 0.459$, two-way ANOVA; NAc-ensemble vs. NAc-ensemble-D2, wake: $p > 0.99$, NREM: $p = 0.351$, REM: $p > 0.99$; Bonferroni's multiple comparisons test). Data are presented as median (red line) with 25th and 75th percentile (dash line). **e** The distributions of both Wake-NREM and REM-NREM modulations were similar between NAc-ensemble neurons and NAc-ensemble-D2 neurons. **f** Mean firing rates of identified NAc-ensemble-D2 neurons (blue, n = 29 units from 5 mice) during different brain state transitions. Data are mean ± SEM. Shading represents ± SEM. ns, not significant. *** $p < 0.001$, ns, not significant. Two-way ANOVA test with Bonferroni's multiple comparisons test for (**d**).

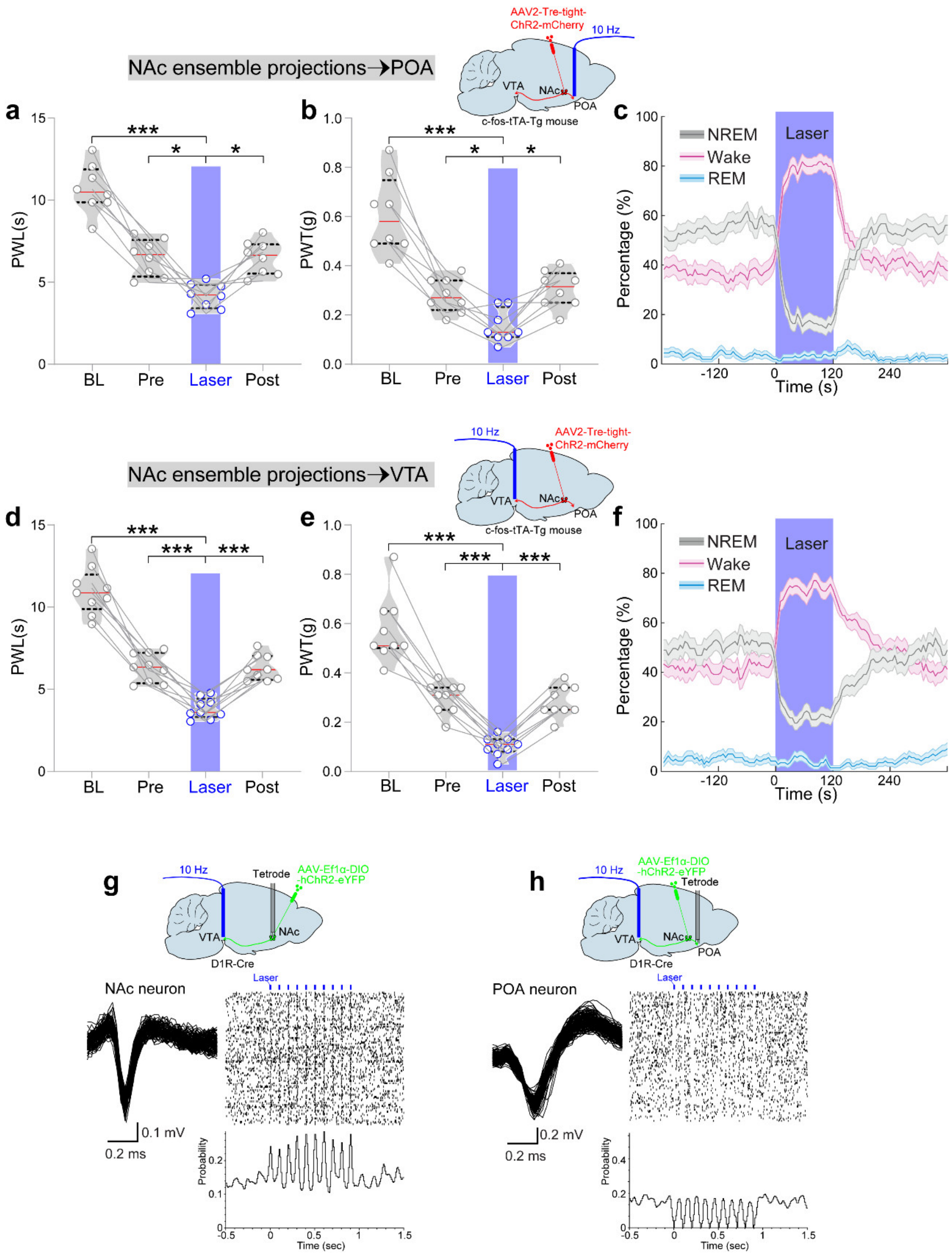

Supplementary Fig. 13 Optogenetic activation of NAc ensemble to POA or VTA projections reduces both pain thresholds and NREM sleep.

a, b Optogenetic activation (473 nm, 10 Hz) of NAc ensemble to POA projections significantly decreases thermal PWL (n = 8 mice, $F_{(2.202, 15.41)} = 54.13$, $p < 0.0001$, BL vs. Laser, p = 0.0001, Pre vs. Laser, p = 0.041, Post vs. Laser, p = 0.0152) and

mechanical PWT (n = 8 mice, $F_{(1.604, 11.23)} = 40.59$, $p < 0.0001$, BL vs. Laser, p = 0.0008, Pre vs. Laser, p = 0.0213, Post vs. Laser, p = 0.0118) in c-fos-tTA-Tg mice. **c** Percentage of time in different brain states before, during, and after blue laser activation of NAc ensemble to POA projections (473 nm, 10 Hz, 120 s; n = 8 mice). Note the dramatical decrease in NREM sleep ($p < 0.0001$, bootstrap), increase in wakefulness ($p < 0.0001$) no obvious change in REM sleep during laser stimulation ($p > 0.05$), n = 8 mice. Data are mean ± SEM. Shading represents ± SEM. **d, e** Optogenetic activation (473 nm, 10 Hz) of NAc ensemble to VTA projections dramatically decreases thermal PWL (n = 9 mice, $F_{(1.821, 14.57)} = 104.8$, $p < 0.0001$, BL vs. Laser, $p < 0.0001$, Pre vs. Laser, $p < 0.001$, Post vs. Laser, $p < 0.001$) and mechanical PWT (n = 9 mice, $F_{(1.385, 11.08)} = 73.15$, $p < 0.0001$, BL vs. Laser, $p < 0.0001$, Pre vs. Laser, $p < 0.001$, Post vs. Laser, $p < 0.001$) in c-fos-tTA-Tg mice. **f** Percentage of time in different brain states before, during, and after blue laser activation of NAc ensemble to VTA projections (473 nm, 10 Hz, 120 s; n = 9 mice). Note the significant decrease in NREM sleep ($p < 0.0001$, bootstrap), increase in wakefulness ($p < 0.0001$) no obvious change in REM sleep during laser stimulation ($p > 0.05$), n = 9 mice. Data are mean ± SEM. Shading represents ± SEM. **g** Top: Schematic drawing of configuration for optogenetic activation of NAc D1 neuron terminals in VTA and tetrode recording at the soma of D1 neurons in NAc. Bottom: Raster plot and PSTH of recording at the soma of a NAc D1 neuron showing the firing activity before, during, and after 10Hz terminal activation in VTA. **h** Top: Schematic drawing of configuration for optogenetic activation of NAc D1 neuron terminals in VTA and tetrode recording at the soma of POA neurons. Bottom: Raster plot and PSTH of recording at the soma of a POA neuron showing the firing activity before, during, and after 10Hz terminal activation in VTA. Brain figures in (**b, e, g, h**) were adapted from Allen Mouse Brain Atlas (2011 Allen Institute for Cell Science, Allen Mouse Brain Atlas, available at http://atlas.brain-map.org/). *** $p < 0.001$, * $p < 0.05$. Repeated measures one-way ANOVA test with Bonferroni's multiple comparisons test for (**a, b, d, e**).

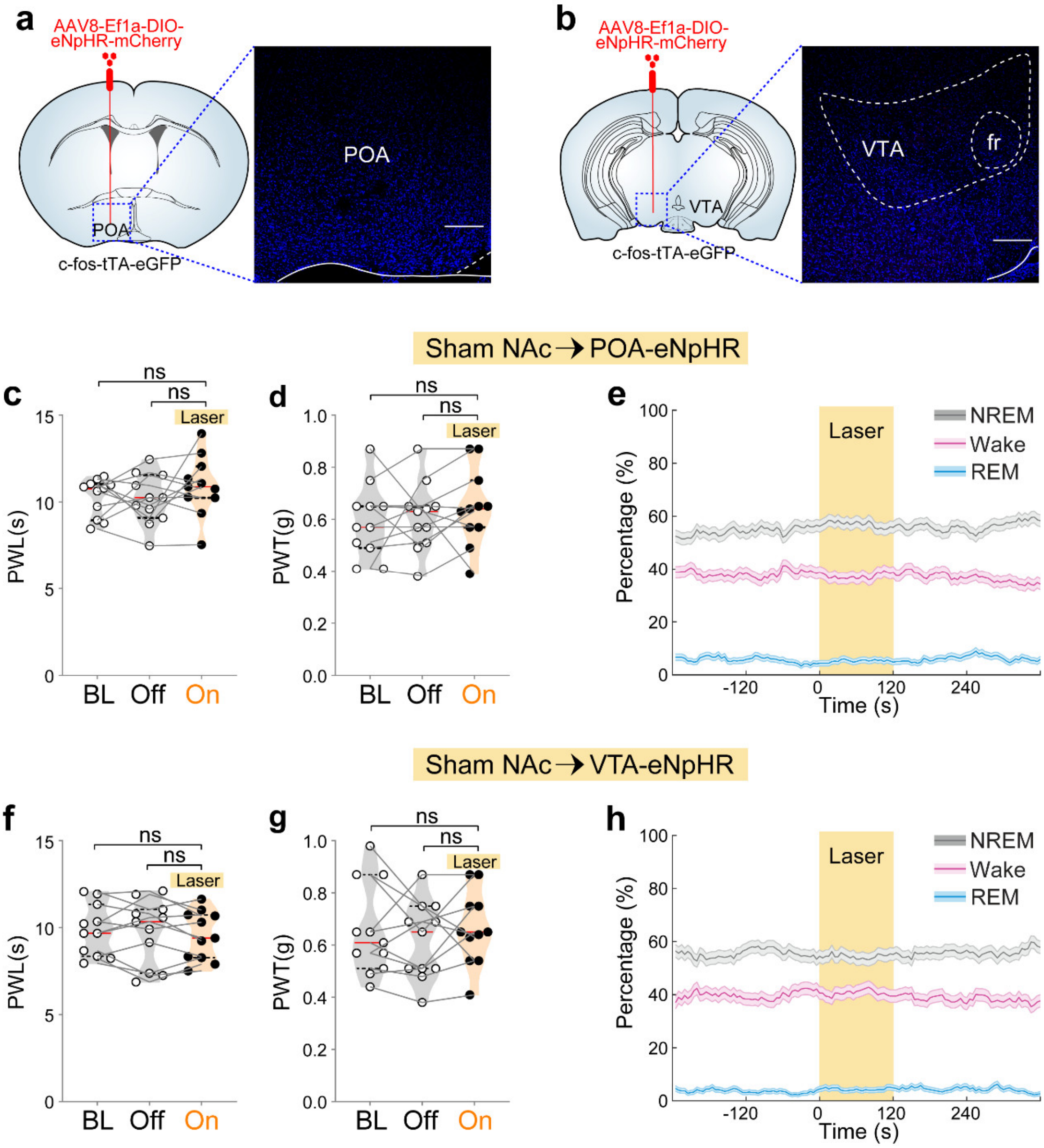

Supplementary Fig. 14 No effect of inactivating POA neurons or VTA neurons innervated by NAc neurons in sham mice on pain thresholds and sleep-wake behavior.

a Schematic showing the injection of AAV8-Ef1a-DIO-eNpHR-mCherry only into POA of c-fos-tTA transgenic mice underwent CCI surgery. Right: a representative segment showing the overlap between DAPI and red-fluorescence in POA. Scale bar: 200 μm. Note that no mCherry was expressed in POA without injection of pAAV1-PTRE-tight-NLS-Cre into NAc. **b** Similar to panel **a**, but for the injection into VTA in CCI mice. Brain figures in (**a** and **b**) were adapted from Allen Mouse Brain Atlas (2011

Allen Institute for Cell Science, Allen Mouse Brain Atlas, available at http://atlas.brain-map.org/). **c-h** The procedure was the same as Fig. 7**a**, but for the c-fos-tTA transgenic mice underwent sham surgery. **c**, **d** No significant differences in thermal pain threshold (PWL, n = 11 mice, $F_{(1.64, 16.4)} = 1.48$, p = 0.254, Repeated measures one-way ANOVA test; BL vs. On, p = 0.29, Off vs. On, p = 0.61, Bonferroni's multiple comparisons test) and mechanical pain threshold (PWT, n = 11 mice, $F_{(1.41, 14.17)} = 0.55$, p = 0.528, Repeated measures one-way ANOVA test; BL vs. On, p > 0.99, Off vs. On, p > 0.99, Bonferroni's multiple comparisons test) of inactivating POA neurons are observed in sham mice between with and without laser inactivations (yellow shading, 589 nm, 8 s on/2 s off). ns, not significant. **e** No effect of yellow laser inactivation of POA neurons (yellow shading, 589 nm, 8 s on/2 s off,120 s) on brain states in sham mice (n = 11 mice, p = 0.30, = 0.21, and = 0.25 for NREM, wake, and REM states). Data are mean ± SEM. Shading represents ± SEM. **f**, **g** No significant differences in thermal pain latency (PWL, n = 11 mice, $F_{(1.97, 19.7)} = 0.62$, p = 0.54, Repeated measures one-way ANOVA test; BL vs. On, p = 0.84, Off vs. On, p > 0.99, Bonferroni's multiple comparisons test) and mechanical pain threshold (PWT, n = 11 mice, $F_{(1.65, 16.5)} = 0.56$, p = 0.55, Repeated measures one-way ANOVA test; BL vs. On, p > 0.99, Off vs. On, p = 0.87, Bonferroni's multiple comparisons test) of inactivating VTA neurons are observed in sham mice between with and without laser inactivations (yellow shading, 589 nm, 8 s on/2 s off). **h** No effect of yellow laser inactivation of VTA neurons (yellow shading, 589 nm, 8 s on/2 s off,120 s) on brain states in sham mice (n = 11 mice, p = 0.254, = 0.209, and = 0.249 for NREM, wake, and REM states). Data are mean ± SEM. Shading represents ± SEM. ns, not significant. Repeated measures one-way ANOVA test with Bonferroni's multiple comparisons test for (**c, d, f, g**).
