# Supplementary material for: A common neuronal ensemble in nucleus accumbens regulates pain-like behaviour and sleep
Source: Nat Commun. 2023 Aug 5;14:4700. doi: 10.1038/s41467-023-40450-3 (PMC10404280; doi:10.1038/s41467-023-40450-3)
Supplement: Supplementary file 3 — Reporting Summary [file 41467_2023_40450_MOESM3_ESM.pdf]

nature portfolio

Corresponding author(s): Jun-Li Cao, Shuming An

Last updated by author(s): Jul 7, 2023

Reporting Summary

Nature Portfolio wishes to improve the reproducibility of the work that we publish. This form provides structure for consistency and transparency in reporting. For further information on Nature Portfolio policies, see our Editorial Policies and the Editorial Policy Checklist.

Statistics

For all statistical analyses, confirm that the following items are present in the figure legend, table legend, main text, or Methods section.

n/a	Confirmed	
☐	☒	The exact sample size (*n*) for each experimental group/condition, given as a discrete number and unit of measurement
☐	☒	A statement on whether measurements were taken from distinct samples or whether the same sample was measured repeatedly
☐	☒	The statistical test(s) used AND whether they are one- or two-sided<br>*Only common tests should be described solely by name; describe more complex techniques in the Methods section.*
☒	☐	A description of all covariates tested
☐	☒	A description of any assumptions or corrections, such as tests of normality and adjustment for multiple comparisons
☐	☒	A full description of the statistical parameters including central tendency (e.g. means) or other basic estimates (e.g. regression coefficient) AND variation (e.g. standard deviation) or associated estimates of uncertainty (e.g. confidence intervals)
☐	☒	For null hypothesis testing, the test statistic (e.g. *F*, *t*, *r*) with confidence intervals, effect sizes, degrees of freedom and *P* value noted<br>*Give P values as exact values whenever suitable.*
☒	☐	For Bayesian analysis, information on the choice of priors and Markov chain Monte Carlo settings
☒	☐	For hierarchical and complex designs, identification of the appropriate level for tests and full reporting of outcomes
☒	☐	Estimates of effect sizes (e.g. Cohen's *d*, Pearson's *r*), indicating how they were calculated

Our web collection on statistics for biologists contains articles on many of the points above.

Software and code

Policy information about availability of computer code

Data collection	1. Electrophysiological data were collected with a Multi-channel Neurophysiology Recording System and NeuroLego amplifier (Jiangsu Brain Medical Technology Co.ltd, Nanjing, China).<br>2. Fluorescence imaging data were collected with a Zeiss LSM 880 confocal microscope and Zen2 software.<br>3. Custom codes are available on GitHub (https://github.com/brain-state-analysis/-Sun-2023_Nature-Communications.git).
Data analysis	1. We used GraphPad 8.0, SigmaPlot 14.0, MATLAB R2017a, NeuroExplorer (v_5.0 & v_5.2), SPSS Statistics V22.0, and Offline Sorter V3 for data analysis in this manuscript.<br>2. Immunofluorescence data were analyzed with Fiji-ImageJ (V_1.52a).

For manuscripts utilizing custom algorithms or software that are central to the research but not yet described in published literature, software must be made available to editors and reviewers. We strongly encourage code deposition in a community repository (e.g. GitHub). See the Nature Portfolio guidelines for submitting code & software for further information.

Data

Policy information about availability of data

All manuscripts must include a data availability statement. This statement should provide the following information, where applicable:

- Accession codes, unique identifiers, or web links for publicly available datasets
- A description of any restrictions on data availability
- For clinical datasets or third party data, please ensure that the statement adheres to our policy

All data in the main text and the supplementary materials are available from the corresponding authors upon request. Source data for Figs. 1 – 8 and Supplementary Figs. 1–14 are provided in this paper. Because further raw data are huge and presented in highly diverse nature and formats, these raw data are available from the corresponding author upon reasonable request. Source data are provided as a Source Data file. Source data are provided with this paper.

Research involving human participants, their data, or biological material

Policy information about studies with human participants or human data. See also policy information about sex, gender (identity/presentation), and sexual orientation and race, ethnicity and racism.

Reporting on sex and gender	N/A
Reporting on race, ethnicity, or other socially relevant groupings	N/A
Population characteristics	N/A
Recruitment	N/A
Ethics oversight	N/A

Note that full information on the approval of the study protocol must also be provided in the manuscript.

Field-specific reporting

Please select the one below that is the best fit for your research. If you are not sure, read the appropriate sections before making your selection.

☒ Life sciences ☐ Behavioural & social sciences ☐ Ecological, evolutionary & environmental sciences

For a reference copy of the document with all sections, see nature.com/documents/nr-reporting-summary-flat.pdf

Life sciences study design

All studies must disclose on these points even when the disclosure is negative.

Sample size	The number of mice used in each experiment was calculated in a priori power analysis (StatMate 2.0), the power of each experiment was set to 0.8.
Data exclusions	We confirmed the viral expression and location of the tips of optical fibers in mice after behavioral tests, and excluded data from mice with mis-targeted viral injection and expression, and optical fiber implants.
Replication	We let two performers test the same groups of mice separately. The attempts at replication were successful based on at least two independent experiments. Behavioral experiments were performed at least twice in individual mouse more than 1 week apart.
Randomization	We randomly assigned 25-29 g mice (3-5 months old) into different groups for all experiments.
Blinding	The investigators were blinded to group allocation during data collection and analysis. The experimenters performing behavioral assays did not know the viral vectors transfected for optogenetic modulation. The analyses of behavioral data were performed blind to the conditions of experiments as data obtained under different conditions were pooled together for automatic batch analysis with computer software.

Reporting for specific materials, systems and methods

We require information from authors about some types of materials, experimental systems and methods used in many studies. Here, indicate whether each material, system or method listed is relevant to your study. If you are not sure if a list item applies to your research, read the appropriate section before selecting a response.

Materials & experimental systems

n/a	Involved in the study
☒	☐ Antibodies
☒	☐ Eukaryotic cell lines
☒	☐ Palaeontology and archaeology
☐	☒ Animals and other organisms
☒	☐ Clinical data
☒	☐ Dual use research of concern
☒	☐ Plants

Methods

n/a	Involved in the study
☒	☐ ChIP-seq
☒	☐ Flow cytometry
☒	☐ MRI-based neuroimaging

Animals and other research organisms

Policy information about studies involving animals; ARRIVE guidelines recommended for reporting animal research, and Sex and Gender in Research

Laboratory animals	Male and female c-fos-tTA mice (stock number: 018306) were obtained from The Jackson Laboratory. Male and female D1R-Cre ((B6.FVB(Cg)-Tg(Drd1a-Cre) EY266Gsat/Mmucd) and D2R-Cre (B6.FVB(Cg)-Tg(Drd2-cre) ER44Gsat/Mmucd) mice were obtained from the GENSAT. Male and female C57/BL6J mice were obtained from the Laboratory Animal Center at Xuzhou Medical University. All mice (3-5 months old) were housed in an environment with a constant temperature of 22 ± 1 °C, humidity of 50 ± 1%, and under12 hr light/12 hr dark cycle (light on from 7 a.m. to 7 p.m.) with ad libitum food and water. The care and use of animals and the experimental protocols (202207S001) used in this study were approved by the Institutional Animal Care and Use Committee and the Office of Laboratory Animal Resources of Xuzhou Medical University under the Regulations for the Administration of Affairs Concerning Experimental Animals (1988) in China.
Wild animals	The study did not involve wild animals.
Reporting on sex	Both male and female mice were used in the study.
Field-collected samples	The study did not involve data collected from the field.
Ethics oversight	The care and use of animals and the experimental protocols (202207S001) used in this study were approved by the Institutional Animal Care and Use Committee and the Office of Laboratory Animal Resources of Xuzhou Medical University under the Regulations for the Administration of Affairs Concerning Experimental Animals (1988) in China.

Note that full information on the approval of the study protocol must also be provided in the manuscript.
